# A prognostic model for hepatitis B acute‐on‐chronic liver failure patients treated using a plasma exchange‐centered liver support system

**DOI:** 10.1002/jca.21762

**Published:** 2019-11-26

**Authors:** Zhongyang Xie, Laurencia Violetta, Ermei Chen, Kaizhou Huang, Daxian Wu, Xiaowei Xu, Xiaoxi Ouyang, Yalei Zhao, Lanjuan Li

**Affiliations:** ^1^ State Key Laboratory for Diagnosis and Treatment of Infectious Diseases, The First Affiliated Hospital, College of Medicine Zhejiang University Hangzhou China; ^2^ Collaborative Innovation Center for Diagnosis and Treatment of Infectious Diseases Zhejiang University Hangzhou China; ^3^ Department of Infectious Disease, The First Affiliated Hospital, College of Medicine Zhejiang University Hangzhou China

**Keywords:** AFP, ALSS, HBV‐ACLF, plasma exchange

## Abstract

**Aim:**

To determine the prognostic risk factors of patients with hepatitis B virus related acute‐on‐chronic liver failure (HBV‐ACLF) treated with plasma exchange (PE)‐based artificial liver support system (ALSS), and create a prognostic predictive model.

**Methods:**

A total of 304 HBV‐ACLF patients who received PE‐based ALSS were retrospectively analyzed. Potential prognostic factors on admission associated with survival were investigated. Of note, 101 additional patients were analyzed to validate the performance of the prognostic models.

**Results:**

According to 28‐day survival, a total of 207 patients who survived and 97 non‐survivors were identified in the derivation group. Overall, 268 (88.2%) ACLF cases were caused by reactivation of HBV. Cox proportional hazards regression model revealed that age, total bilirubin, ln (alpha‐fetoprotein [AFP]), encephalopathy (HE) score, sodium level, and international normalized ratio (INR) were independent risk factors of short‐term prognosis. We built a model named ALSS‐prognosis model (APM) to predict the 28‐day survival of HBV‐ACLF patients with ALSS; the model APM showed potentially better predictive performance for both the derivation and validation groups than MELD, MELD‐Na, and CLIF‐C ACLF score.

**Conclusions:**

Low AFP was found to be an independent risk factor for high mortality in HBV‐ACLF patients treated with PE‐based ALSS. We generated a new model containing AFP, namely APM, which showed potentially better prediction performance than MELD, MELD‐Na, and CLIF‐C ACLF score for short‐term outcomes, and could aid physicians in making optimal therapeutic decisions.

## 1 INTRODUCTION

With an estimated 130 million carriers and 30 million chronically infected individuals, China has been recognized as a high‐prevalence area for hepatitis B virus (HBV).^1^ Individuals with chronic HBV are at risk of developing an acute exacerbation leading to acute‐on‐chronic liver failure (ACLF), which has high short‐term mortality.^2^ Liver transplantation (LT) has been associated with long‐term survival in these patients. However, donor liver scarcity and socioeconomic challenges have made LT impracticable, thus alternative therapies are required to overcome this unmet need.^2^ Since the late fifties, a variety of artificial liver support systems (ALSSs) have been utilized to treat liver failure. Several studies have shown that ALSSs, in particular plasma exchange (PE), prolong the survival of patients with both ALF and ACLF.^3^, ^4^, ^5^ In addition to rapid elimination of toxic metabolites, PE is also able to replenish beneficial plasma factors, which serves as a bridge to LT by providing a suitable environment for liver recovery.^6^

An accurate prognostic scoring system is necessary to estimate disease severity and accurately appoint ACLF patients to the appropriate management. Various prognostic models including the Model for End‐stage Liver Disease (MELD),^7^ MELD‐sodium (MELD‐Na),^8^ and Chronic Liver Failure‐Consortium (CLIF‐C) ACLF^9^ score were deemed suitable to predict severity in ACLF patients with HBV etiology.^9^, ^10^ Conversely, due to clinical discrepancies between Eastern and Western ACLF,^11^ these scores were found to be less sensitive for early diagnosis of HBV‐ACLF compared to the score recently proposed score by the Study of Severe Hepatitis B (COSSH)^12^ in China, which was constructed based on a large, multicenter cohort of HBV‐related ACLF patients. However, whether these models can be used to evaluate the prognosis of HBV‐ACLF patients with ALSS is unknown. For patients receiving ALSS treatment, the factors related to prognostic and curative effects need to be analyzed.

In the present study, we investigated the prognosis, and factors affecting prognosis, in patients with HBV‐ACLF treated using PE‐based ALSS. Furthermore, we propose a novel model that could predict prognosis in these patients.

## 2 MATERIALS AND METHODS

### 2.1 Study design

Liver failure patients who received ALSS at our hospital between January 2015 and July 2017 were identified retrospectively. The operating guide for ALSSs was strictly applied to identify patients for ALSS treatment.^13^ Data of all patients were extracted from the electronic medical records system and analyzed anonymously according to the Declaration of Helsinki. This study was approved by the Human Ethics Committee of the First Affiliated Hospital of Zhejiang University.

**Figure 1:**
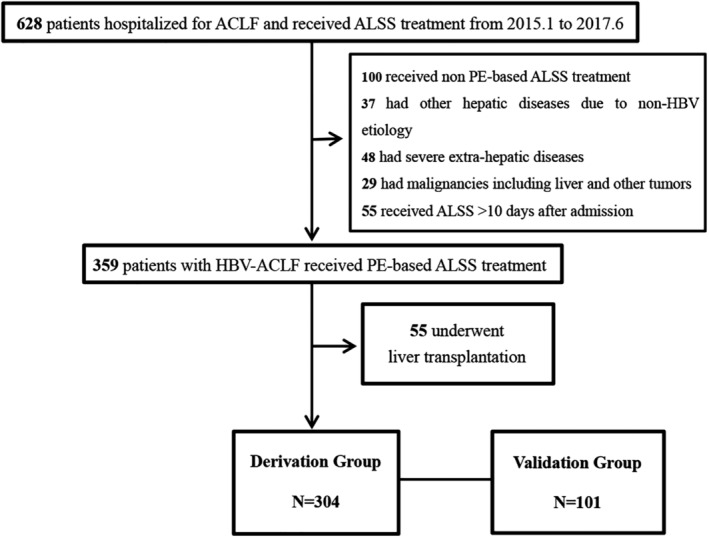
Flow chart of inclusion of HBV‐ACLF patients with ALSS. HBV‐ACLF, HBV related acute‐on‐chronic liver failure; ALSS, artificial liver support system; PE, plasma exchange; LT, liver transplantation

In the derivation group, a total of 628 patients with chronic hepatitis B (CHB),^11^ with or without cirrhosis, who met the diagnostic criteria for ACLF were included in the study (Figure 1). Patients with liver failure of non‐HBV etiology or co‐infection with an additional hepatitis virus (n = 37), malignancies (n = 29), non‐PE‐based ALSS (n = 100), or severe extra‐hepatic diseases (n = 49) were excluded from the study. In addition, patients (n = 55) who received the first ALSS treatment more than 1 week after admission were excluded. During the course of hospitalization, 55 patients received LT and the remaining 304 patients were enrolled in this study. Our study also included patients who presented without ACLF at admission, but who later developed ACLF prior to the start of ALSS therapy.

Additional 101 patients were enrolled in the validation group between August 2017 and December 2017 from First Affiliated Hospital of Zhejiang University and Shulan (Hangzhou) Hospital; all of them met the aforementioned inclusion and exclusion criteria.

### 2.2 Definition and staging

As proposed by the Asian Pacific Association for the Study of the Liver,^14^ ACLF was defined as acute liver injury (TB ≥5 mg/dL and INR ≥1.5) complicated within 4 weeks by ascites and/or hepatic encephalopathy (HE) in patients with previously diagnosed or undiagnosed chronic liver disease.

Assessment of different prognostic models was performed according to different criteria and researches.^7^, ^8^, ^12^, ^15^ Cirrhosis was diagnosed based on previous liver biopsy results, clinical evidence of previous decompensation, laboratory tests, endoscopy (esophageal and gastric varices), and radiological evidence of portal hypertension and/or liver nodularity. Ascites was confirmed by abdominal imaging, paracentesis, and clinical evidence of prior decompensation. West Haven criteria were used for the assessment and grading of HE.^16^ Gastrointestinal (GI) bleeding was defined as the presence of blood in the stool or vomit. Common bacterial infections include spontaneous bacterial peritonitis, pulmonary infection, and urinary tract infection.^17^

### 2.3 Data collection

The baseline characteristics recorded consisted of encephalopathy score (determined clinically), plus laboratory parameters including alanine aminotransferase (ALT), aspartate aminotransferase (AST), total bilirubin (TB), albumin, ferritin, alpha‐fetoprotein (AFP), sodium, glucose, creatinine, international normalized ratio (INR), and white blood cell (WBC), hemoglobin, and platelet counts. Furthermore, calculation of MELD, MELD‐Na, CLIF‐C ACLF, and COSSH‐ACLF scores was performed.^7^, ^12^, ^15^ All data were collected at the time of admission. HBV serology and HBV‐DNA were assayed at admission. Any adverse reactions related to ALSS treatment were recorded.

### 2.4 Treatment

Circulatory access was established via the patients' femoral vein and PE was performed using the EC‐40W membrane separation method (Asahi KASEI Co., Tokyo, Japan). The total exchange volume was 2500 to 3500 mL, which composed 1500 to 2500 mL of fresh frozen plasma (FFP) and 500 to 1000 mL of 5% albumin. FFP was provided by the local Blood Transfusion Service, which was obtained by voluntary blood donation. The PE rate was set to 20 to 25 mL/min with an adjusted blood flow rate of 100 to 130 mL/min. Five milligram of dexamethasone was injected routinely to prevent allergic reactions. Based on the prothrombin time prior to PE, approximately 20 to 60 mg of heparin and 10 to 30 mg of protamine sulfate were administered every session. PE‐centered ALSS included the utilization of PE alone or in combination with hemofiltration (HF) or plasma perfusion (PP). HF was performed following PE, which used a BLS816G column (SORIN Group, Como, Italy) at a filtrate flow rate of 50 mL/kg/h for 6 to 8 hours. PP was performed using EC‐40W for plasma separation and two adsorption columns (BS330 and HA330‐II; Jian Fan, Zhuhai, China) for toxin removal. The methods were performed according to patient condition. HF is suitable for encephalopathy, acute kidney injury, and water‐electrolyte imbalance. Bilirubin adsorption is utilized for hyperbilirubinemia with mild coagulation dysfunction (INR <2). ALSS treatment was performed every 2 to 3 times per week and was discontinued if bleeding or circulatory complications occurred.^18^ In total, 873 sessions of ALSS treatment were performed, with an average of two sessions per patient (range: 1‐6 sessions per patient).

All patients received standard medical therapy (SMT), including bed rest, adequate nutritional support, and oral antiviral drugs. Complications were also treated, as follows: ascites were treated with sodium and water restriction and/or diuretics, and peritoneocentesis combined with intravenous albumin when necessary; broad‐spectrum antibiotics were given initially to patients with bacterial infections and later modified based on culture and/or antibiotic sensitivity results; acute GI bleeding was treated with intravenous somatostatin, pituitrin, proton pump inhibitors, and endoscopic therapy when needed; HE patients received lactulose and l‐ornithine aspartate; fluid replacement was applied in patients with low mean arterial pressure and/or vasoconstrictors in those with circulatory dysfunction; and persistent low‐flow oxygen therapy (2‐4 L/min) was administered to patients with a PaO_2_ < 80 mm Hg, with mechanical ventilation provided when severe respiratory dysfunction occurred.

### 2.5 Statistical analysis

All data were analyzed using the SPSS software (ver. 22.0; SPSS Inc., Chicago, Illinois). Continuous variables were expressed as the mean ± SD or median (p25, p75) and were compared using Student's *t* test, Mann‐Whitney *U* test, or Wilcoxon rank‐sum test, and categorical variables by χ^2^ test. Survival rates were estimated by Kaplan‐Meier analysis and compared by the log‐rank test. Patients who received LT were censored at the time of survival analysis. Potential predictors of prognosis were identified by univariate and multivariate analysis using the Cox proportional hazards regression model. Multivariate logistic regression was used to establish the new model and the performance of the prognostic scores was assessed by comparison of receiver operating characteristic (ROC) curves using the *z* test (Delong's method). A *P* value <.05 was considered statistically significant.

## 3 RESULTS

### 3.1 Study population

Table 1 summarized the clinical characteristics of derivation (n = 304) and validation groups (n = 101) of HBV‐ACLF treated with PE‐based ALSS. In the presented cohort, majority of the patients had HBV reactivation (88.2% in derivation group, 89% in validation group) as the cause of ACLF due to spontaneous reactivation, cessation of nucleoside analogs (NUCs) or development of antiviral resistance in patients who had previously started oral NUCs. There was no significant difference in gender distribution, risk factors, the presence of cirrhosis, precipitating events, HBV‐DNA level, ascites, CLIF‐C ACLF score, and majority of the lab parameters at baseline between the two groups.

**Table 1:** Clinical characteristics of derivation and validation groups of HBV‐ACLF patients treated with PE‐based ALSS at admission

	Derivation group (n = 304)	Validation group (n = 101)	*P* value
Age	47.77 ± 11.32	42.88 ± 12.073	<.0001
Gender (female/male)	36/268	11/90	.796
Hypertension	40 (13.2)	14 (13.9)	.857
Diabetes	24 (7.9)	5 (5.0)	.321
Alcohol	76 (25.0)	29 (28.7)	.461
Smoking	115 (37.8)	32 (31.7)	.266
Cirrhosis	128 (42.1)	48 (47.5)	.3432
Precipitating event			
HBV‐reactivation	268 (88.2)	90 (89.0)	.741
Spontaneous reactivation	211 (69.4)	68 (67.3)	
NUC cessation	41 (13.5)	16 (15.8)	
NUC resistance	16 (5.3)	6 (5.9)	
Infection	34 (11.2)	8 (7.9)	
Others	2 (0.7)	3 (3.0)	
HBeAg positive	124 (40.8)	50 (49.5)	.126
HBeAb positive	198 (65.1)	50 (49.5)	.005
Lg(HBV‐DNA)	5 (3, 6)	4 (3, 6)	.583
Complications			
HE	25 (8.2)	27 (26.7)	<.0001
Ascites	88 (36.2)	56 (55.4)	.24
GI bleeding	9 (3.0)	4 (4.0)	<.0001
Bacterial infection	39 (16)	34 (33.7)	.007
MELD score	22.71 (19.8, 25.56)	23.9 (21.5, 28.06)	.006
MELD‐Na score	23.04 (20.3, 26.28)	24.29 (21.65, 29)	.009
CLIF‐C ACLF score	40.11 (36.18, 43.9)	39.89 (35.4, 45.98)	.468
COSSH ACLF score	5.97 (5.51, 6.48)	6.14 (5.65, 6.93)	.049
Laboratory data			
ALT (U/L)	462 (211, 956)	271 (118, 718)	.164
AST (U/L)	313 (137, 639)	215 (93, 456)	.385
TB (μmol/L)	19.33 (15.5, 25.63)	20.47 (15.64, 23.8)	.744
ALP(U/L)	138 (115, 166)	127 (113, 149)	.015
GGT (U/L)	86 (63, 130)	88 (58, 133)	.96
Albumin (g/dL)	32.2 (29.8, 35.1)	33.2 (30.8, 35.6)	.011
Sodium (mmol/L)	138 (136, 140)	138 (135.5, 140)	.834
Creatinine (μmol/L)	0.72 (0.62, 0.85)	0.70 (0.60, 0.83)	.157
INR	1.99 (1.7, 2.54)	2.39 (1.94, 2.91)	<.0001
WBC (10^9^/L)	6.8 (5.4, 9.1)	7 (5.6, 8.9)	.068
Hemoglobin (g/L)	137.5 (123.25, 149)	133 (116, 143)	.348
Platelet count (10^9^/L)	124 (87, 157)	111 (82, 155)	.556
Ferritin (μg/L)	3196 (1705, 6438)	2805 (1499, 5375)	.894
Alpha fetoprotein (μg/L)	95 (38, 245)	88 (35, 261)	.763

*Note*: Data are expressed as mean ± SD, median with interquartile range (p25, p75) or number of patients (percentages). ALP, phosphatase alkaline; ALT, alanine aminotransferase; AST, aspartate aminotransferase; BUN, blood urea nitrogen; CLIF‐C ACLF, European Association for the Study of Chronic Liver Failure; COSSH‐ACLF, Chinese Group on the Study of Severe Hepatitis B; GGT, gamma‐glutamyl transpeptidase; GI, gastrointestinal; HE, hepatic encephalopathy; INR, international normalized ratio; MELD, Model for End‐stage Liver Disease; NUC, nucleoside analogs; WBC, white blood cell count.

The age (*P* < .0001), number of HBeAb positive patients (*P* = .005), presence of GI bleeding (*P* < .0001), bacterial infection (*P* = .007), and level of ALP (*P* = .015) were significantly higher in the derivation group than in the validation group. Meanwhile, the number of patients presented with HE (<.0001), the MELD (*P* = .006), MELD‐Na score (*P* = .009) and COSSH‐ACLF score (*P* = .049), albumin level (*P* = .011), and INR value (*P* < .0001) were significantly less in the derivation group compared to the validation group.

### 3.2 Analysis of prognostic factors of HBV‐ACLF patients with PE‐based ALSS

According to survival at day 28, patients were allocated into survival and non‐survival groups (Table S1). Patients in the survivor group were significantly younger, had significantly less incidence of HE (*P* < .0001), had substantially lower levels of TB (*P* < .001), INR (*P* < .001), D‐dimer (*P* < .001), ferritin (*P* = .015) and WBC counts (*P* < .001), as well as lower MELD, MELD‐Na, CLIF‐C ACLF and COSSH‐ACLF scores (all *P* < .0001), but had higher AFP levels (*P* < .001) at admission compared to HBV‐ACLF patients who did not survive.

Table 2 showed that by univariate analysis, the age, hypertension, HE score, TB, ln(AFP), ln(ferritin), BUN, sodium, INR, WBC count, and all prognostic scores (MELD, MELD‐Na, CLIF‐C ACLF, COSSH‐ACLF) were significantly associated with mortality. Among them, AFP and ferritin were identified as new prognostic markers. Correlation analysis revealed a negative correlation between AFP and AST levels (*r* = −.219; *P* = .0001; Figure S1A) and a positive correlation between ferritin with ALT (*r* = .334, *P* < .001) and AST levels (*r* = .309, *P* < .001; Figure S1B,C). Furthermore, multivariate analysis revealed that age, HE score, TB, ln(AFP), sodium, and INR were independently associated with prognosis at day 28.

**Table 2:** Univariate and multivariate analysis between survival and non‐survival groups

	Univariate Cox regression				Multivariate Cox regression			
Variables	B	SE	HR (95% CI)	*P*	B	SE	OR (95% CI)	*P*
Clinical characteristics								
Gender	0.081	0.308	1.085 (0.593‐1.985)	.792				
Age	0.023	0.009	1.024 (1.006‐1.042)	.010	0.030	0.009	1.03 (1.012‐1.049)	.001
Alcohol	0.149	0.207	1.161 (0.774‐1.742)	.471				
Hypertension	0.539	0.256	1.715 (1.038‐2.832)	.035				
Diabetes	0.428	0.320	1.535 (0.819‐2.875)	.181				
Cirrhosis	−0.002	0.206	0.998 (0.667‐1.494)	.994				
Decompensation history	−0.048	0.369	0.954 (0.463‐1.966)	.898				
Ascites	0.243	0.204	1.276 (0.856‐1.901)	.232				
GI bleeding	0.364	0.511	1.439 (0.529‐3.914)	.476				
Infection	0.445	0.261	1.56 (0.935‐2.604)	.089				
HE score	0.973	0.189	2.645 (1.827‐3.829)	<.001	0.834	0.216	2.303 (1.509‐3.515)	<.001
Laboratory parameters								
HBeAg positive	−0.070	0.208	0.933 (0.62‐1.402)	.737				
HBeAb positive	0.093	0.216	1.097 (0.719‐1.676)	.667				
lg(HBV‐DNA)	0.086	0.051	1.09 (0.986‐1.204)	.093				
ALT	0.000	0.000	1.000 (0.9996‐1.0002)	.687				
AST	0.000	0.000	1.000 (0.9996‐1.0003)	.816				
TB	0.054	0.013	1.056 (1.029‐1.083)	<.001	0.087	0.014	1.091 (1.062‐1.120)	<.001
ALB	0.002	0.003	1.002 (0.995‐1.008)	.633				
ALP	0.003	0.002	1.003 (0.999‐1.007)	.096				
GGT	0.000	0.001	1.000 (0.997‐1.003)	.980				
ln(AFP)	−0.300	0.068	0.741 (0.648‐0.847)	<.001	−0.219	0.077	0.804 (0.691‐0.935)	.005
ln(Ferritin)	0.311	0.117	1.364 (1.086‐1.715)	.008				
Creatinine	0.070	0.179	1.073 (0.756‐1.523)	.694				
BUN	0.020	0.009	1.02 (1.003‐1.038)	.025				
Sodium	−0.076	0.022	0.927 (0.888‐0.967)	.001	−0.045	0.02	0.956 (0.920‐0.993)	.021
INR	1.045	0.154	2.842 (2.100‐3.847)	<.001	0.895	0.16	2.447 (1.788‐3.349)	<.001
FIB	−0.386	0.239	0.680 (0.425‐1.086)	.107				
Hemoglobin	0.005	0.006	1.005 (0.994‐1.016)	.401				
WBC	0.075	0.024	1.077 (1.029‐1.128)	.002				
PLT	−0.001	0.002	0.999 (0.995‐1.003)	.551				
Prognostic score								
MELD	0.134	0.022	1.143 (1.095‐1.193)	<.001				
MELD‐Na	0.091	0.012	1.095 (1.069‐1.122)	<.001				
CLIF‐C ACLF	0.132	0.018	1.141 (1.101‐1.183)	<.001				
COSSH‐ACLF	1.244	0.130	3.47 (2.692‐4.474)	<.001				

Abbreviations: AFP, alpha fetoprotein; ALB, albumin; ALP, phosphatase alkaline; ALT, alanine aminotransferase; AST, aspartate aminotransferase; BUN, blood urea nitrogen; CLIF‐C ACLF, European Association for the Study of Chronic Liver Failure; COSSH‐ACLF, Chinese Group on the Study of Severe Hepatitis B; FIB, fibrinogen; GGT, gamma‐glutamyl transpeptidase; HE, hepatic encephalopathy; INR, international normalized ratio; MELD, Model for End‐stage Liver Disease; PLT, platelets; TB, total bilirubin; WBC, white blood cell count.

**Figure 2:**
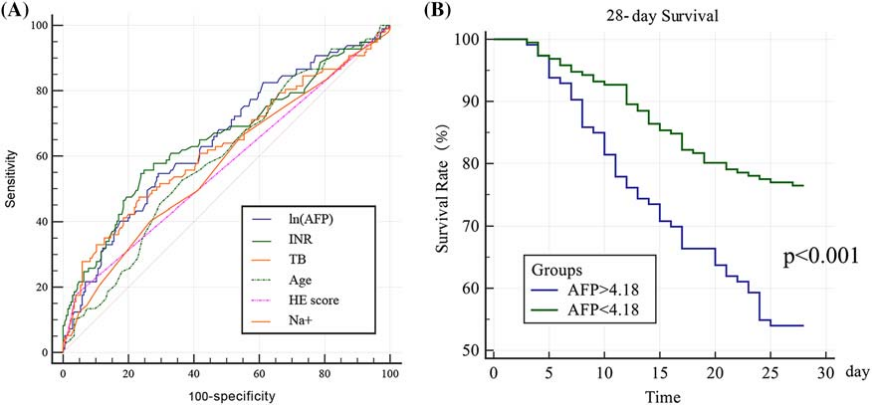
Receiver operating characteristic curves of single prognostic factor (A) and Kaplan–Meier curve of the high—AFP group (ln(AFP) > 4.18) and low—AFP group (ln(AFP) ≤ 4.18)

Then, we further analyzed the prognostic value of ln(AFP). ROC curve showed that ln(AFP) had the second largest AUROC, superior to TB, HE score, age, and sodium (Figure 2A and Table 3). According to the cutoff value, patients in the derivation group were divided into low AFP group (ln(AFP) < 4.18) and high AFP group (ln(AFP) > 4.18). Kaplan‐Meier curve revealed that high AFP group had significantly higher 28‐day survival rate than low AFP group (*P* < .001, Figure 2B).

**Table 3:** AUROC of single prognostic factors

Variables	AUROC	SE	95% CI
ln(AFP)	0.649	0.0344	0.593‐0.703
INR	0.653	0.0358	0.596‐0.707
TB	0.627	0.0363	0.570‐0.682
Age	0.592	0.0346	0.535‐0.648
HE score	0.568	0.0205	0.510‐0.625
Sodium	0.575	0.036	0.517‐0.631

Abbreviations: AFP, alpha fetoprotein; AUROC, area under the receiver operating characteristic curves; HE, hepatic encephalopathy; INR, international normalized ratio; TB, total bilirubin.

### 3.3 Establishment and validation of a new prognostic model containing AFP

**Figure 3:**
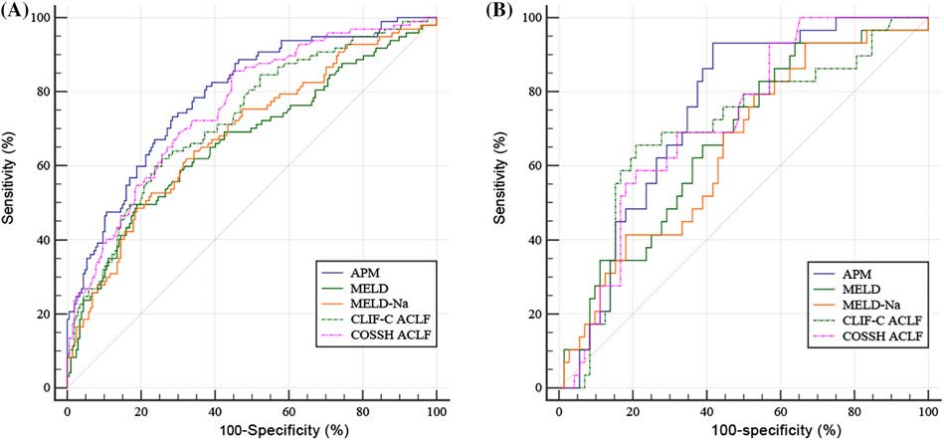
Receiver operating characteristic (ROC) curves illustrating the ability of different prognostic models in derivation (A) and validation (B) groups to predict the 28‐day mortality of HBV‐ACLF patients receiving ALSS. APM: 0.042 × age + TB (mg/dL) × 0.094 + 1.228 × INR−0.473 × ln[AFP (μg/L)]

Using logistic regression model, we generated a new prognostic model, the ALSS‐prognosis model (APM), using a four factor multivariate logistic regression: 0.042 × age + TB (mg/dL) × 0.094 + 1.228 × INR−0.473 × ln[AFP (μg/L)]. The predictive values of the different models are illustrated in Table 4 and Figure 3. APM demonstrated a significantly higher AUROC (0.790) compared to MELD (AUROC = 0.666, *P* = .0003), MELD‐Na (AUROC = 0.685, *P* = .001), and CLIF‐C ACLF (AUROC = 0.726, *P* = .0184), but not compared to COSSH‐ACLF (AUROC = 0.759, *P* = .1292) for the derivation group.

**Table 4:** Comparison of the predictive value of prognostic scoring systems for HBV‐ACLF patients with PE‐based ALSS

Model	Derivation group			Derivation group			Validation group		
	Sensitivity (%)	Specificity (%)	Cutoff value	AUROC	95% CI	*P* value	AUROC	95% CI	*P* value
APM	73.2	71.5	2.56	0.790	0.740‐0.834		0.747	0.651‐0.828	
MELD	49.48	81.16	24.8	0.666	0.610‐0.7219	.0003	0.667	0.566‐0.757	.0648
MELD‐Na	52.58	77.29	24.8	0.685	0.629‐0.736	.001	0.645	0.543‐0.737	.0308
CLIF‐C ACLF	61.86	74.40	41.99	0.726	0.673‐0.776	.0184	0.694	0.595‐0.782	.3367
COSSH‐ACLF	84.54	55.56	5.88	0.759	0.706‐0.806	.1292	0.718	0.620‐0.803	.4948

Abbreviations: APM: 0.042 × age + TB (mg/dL) × 0.094 + 1.228 × INR−0.473 × ln[AFP (μg/L)]. AUROC, area under the receiver operating characteristic curves; MELD, Model for End‐stage Liver Disease; COSSH‐ACLF, Chinese Group on the Study of Severe Hepatitis B; CLIF‐C ACLF, European Association for the Study of Chronic Liver Failure.

The established models were further verified in the validation group. Consistent with the derivation group, APM had the highest AUROC value among all prognostic models. As a result of the small sample size in the verification group, APM was significantly different to MELD‐Na (*P* = .0308), but no significant difference was observed compared to MELD, CLIF‐C ACLF, and COSSH‐ACLF.

## 4 DISCUSSION

Despite the introduction to antivirals, patients with HBV‐ACLF have poor survival owing to HBV reactivation and the development of antiviral resistance.^19^ HBV‐ACLF has become one of the most common indications for LT at our center, but liver donor shortage necessitates the need for alternative therapy. PE has been widely used in China due to its ability to rapidly remove over‐accumulated toxic materials and replace beneficial plasma substances.^20^, ^21^ Although PE alone is useful, combining PE with other purification methods is even more effective.^22^, ^23^ It was reported that if MELD score could be reduced prior LT, the prognosis of HBV‐ACLF could be improved.^24^ Previous studies have reported that treatment with PE‐based ALSS could significantly lowered serum bilirubin, liver enzyme levels and INR, as well as improved MELD score in HBV‐ACLF patients.^5^, ^18^ These effects from PE might help to reduce the metabolic burden and promote the regeneration of hepatocytes, thus prolonging the survival of patients. Xu et al^25^ also demonstrated the importance of ALSS in terms of providing additional time for HBV‐ACLF patients awaiting LT.

Although several previous prognostic models for ACLF have been derived and verified among eastern and western countries, no specific model for prediction of HBV‐ACLF patients who received ALSS treatment has previously been published in any detail. In this study, we used MELD, MELD‐Na, CLIF‐C ACLF, and COSSH‐ACLF to predict the short‐term prognosis on this group of patients. The COSSH‐ACLF^12^ scoring system was established specifically for ACLF patients with HBV etiology, with liver and coagulation failure as the most common types of organ failures, which explained the low serum creatinine values of patients in the current study. Its prognostic value for HBV‐ACLF patients receiving ALSS have been confirmed in our study. This score was consistent with unique characteristics of ACLF that include hyperbilirubinemia (TB), coagulopathy (INR), and multiple OF (systemic organ failure assessment [SOFA]), which enrolled seven evaluation indicators in total. MELD was previously used to evaluate the curative effects of ALSS,^26^ but in our study, the AUROC only reached 0.666, which was the lowest among the models we used for evaluating prognosis.

In this study, we found that high AFP levels were associated with improved outcomes in HBV‐ACLF patients receiving ALSS. AFP has been widely used as a clinical biomarker of chronic liver diseases and hepatic malignancy.^27^ Huang et al^28^ suggested that AFP is a marker of liver regeneration, whereby a high AFP was associated with greater hepatocyte regeneration and favorable outcomes in patients with HBV‐ACLF. Kakisaka et al^29^ suggested that serum AFP level may reflect the induction of liver progenitor cells in acute liver failure patients, and the persistent induction of liver progenitor cells may be needed for a recovery from liver failure. These findings suggested that patients with high AFP had greater regeneration activity and thus a better response to ALSS. Luo et al^30^ combined MELD with AFP, HE, WBC, and age to generate a new prognostic model to predict the prognosis of HBV‐ACLF patients. This model performed a higher AUROC than MELD, MELD‐Na, and iMELD. Nonetheless, the modified MELD as described by Luo et al requires the input of a large number of independent factors, making it complicated and difficult to use in routine clinical practice.

The new model APM was generated to predict the outcome of patients with HBV‐ACLF treated with PE‐based ALSS. It was superior in comparison to the other models for predicting the 28‐day mortality, as demonstrated by the AUROC. With a cutoff value of 2.56, APM significantly surpassed the predictive value of the other models, except for COSSH‐ACLF. Then, we evaluated its performance in a validation group containing 101 patients from two different hospitals. Similarly as in the derivation group, APM performed highest AUROC than other models. Although our APM model did not demonstrate a significant advantage compared to the COSSH‐ACLF scoring system in derivation group, our model only included four factors, making it significantly more convenient to calculate and apply in the clinical setting. Our APM could be a useful tool to assist clinicians in deciding if ALSS treatment is beneficial for a given patient with HBV‐ACLF; ALSS offers minimal benefit to patients with a score above 2.56 and LT should be used for management of those patients.

Our study had some limitations. First, it used a retrospective design and data were only collected for patients with HBV‐ACLF from a single medical center. Second, no comparison was performed with patients who only received conventional medical treatment. Although our new model offered better prognostic prediction efficiency, it can only be applied to HBV‐ACLF patients receiving additional ALSS therapy. Whether this model can be applied to other etiologies of ACLF requires further validation in randomized controlled trials involving multiple centers that include more heterogeneous groups of ACLF patients.

## 5 CONCLUSIONS

In our study, low serum AFP level was found to be an independent risk factor for high mortality in HBV‐ACLF patients with PE‐based ALSS. We have proposed a new prognostic prediction model containing AFP, namely APM, which was superior for predicting short‐term outcomes and may aid physicians in choosing the best therapeutic management method.

## Supporting information


**Figure S1** Correlations of AFP and ferritin with other biochemical indicators. The logarithmic transformed concentrations of AFP were inversely correlated with serum AST (A) (logarithmic transformed). Ferritin was positively correlated with serum ALT (A) (logarithmic transformed) and AST (B). *r*, spearman's correlation coefficient; *P* values indicate the significance of correlations (two‐tailed).

**Table S1** Clinical characteristics of survivors and non‐survivors groups of HBV‐ACLF patients treated with PE‐based ALSS at admission
