# Supplementary figures and images for: A prognostic model for hepatitis B acute‐on‐chronic liver failure patients treated using a plasma exchange‐centered liver support system
Source: J Clin Apher. 2019 Nov 26;35(2):94–103. doi: 10.1002/jca.21762 (PMC7217207; doi:10.1002/jca.21762)

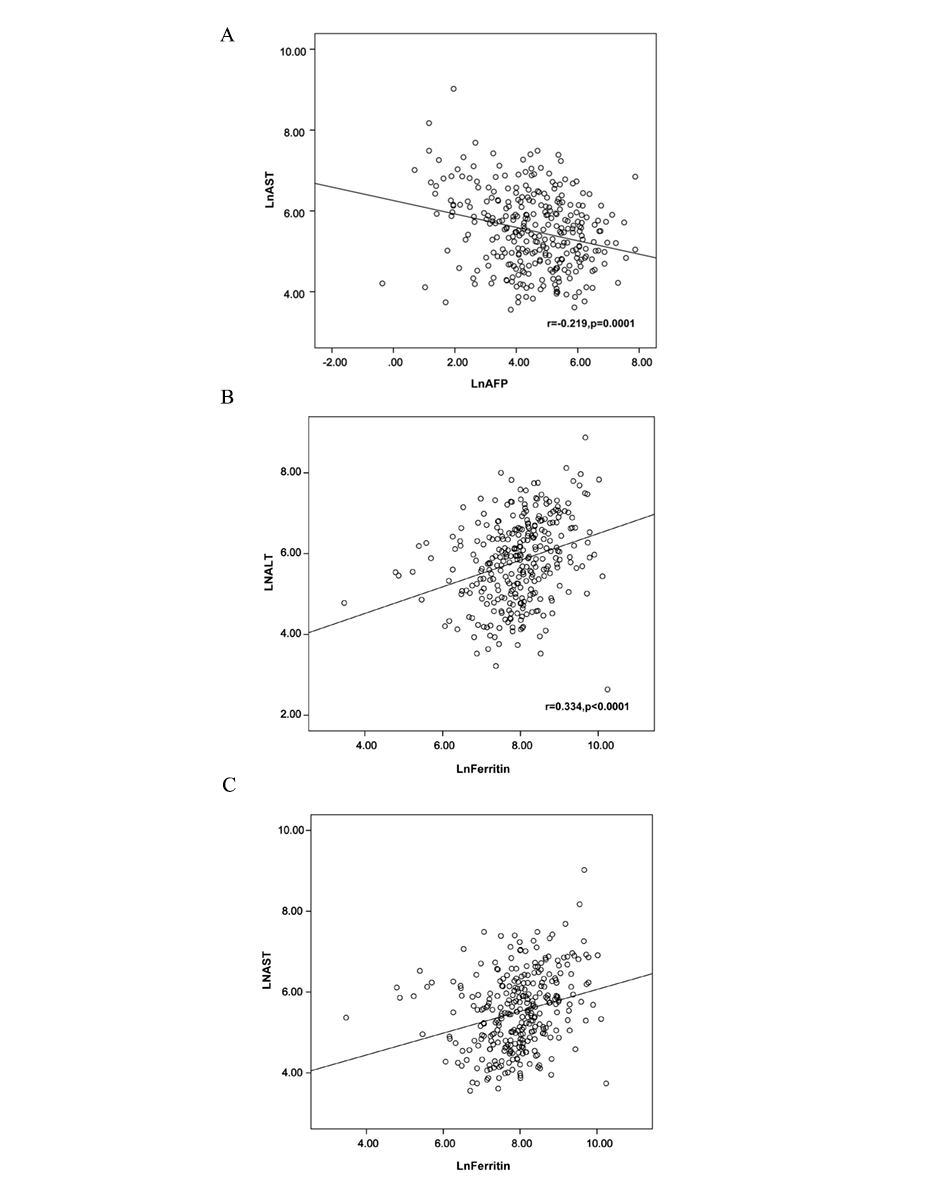

Supplement: Supplementary file 1 — Figure S1 Correlations of AFP and ferritin with other biochemical indicators. The logarithmic transformed concentrations of AFP were inversely correlated with serum AST (A) (logarithmic transformed). Ferritin was positively correlated with serum ALT (A) (logarithmic transformed) and AST (B). r, spearman's correlation coefficient; P values indicate the significance of correlations (two‐tailed). [file JCA-35-94-s001.tif]
