# Supplementary material for: A prognostic model for hepatitis B acute‐on‐chronic liver failure patients treated using a plasma exchange‐centered liver support system
Source: J Clin Apher. 2019 Nov 26;35(2):94–103. doi: 10.1002/jca.21762 (PMC7217207; doi:10.1002/jca.21762)
Supplement: Supplementary file 2 — Table S1 Clinical characteristics of survivors and non‐survivors groups of HBV‐ACLF patients treated with PE‐based ALSS at admission [file JCA-35-94-s002.docx]

**Table S1 Clinical characteristics of survivors and non-survivors groups of HBV-ACLF patients treated with PE-based ALSS at admission**

|  | Survivor  （n=207） | Non-survivor  （n=97） | P value |
| --- | --- | --- | --- |
| Age | 46.61±11.26 | 50.24±11.10 | 0.009 |
| Gender(female/male) | 24/183 | 12/85 | 0.845 |
| Hypertension | 21(10.1) | 19（19.6） | 0.023 |
| Diabetes | 13(6.3) | 11（11.3） | 0.128 |
| Smoking | 115(37.8) | 39（40.2) | 0.559 |
| Cirrhosis | 87(42) | 41(42.3) | 0.969 |
| Prior Decompensation | 18(8.7) | 8(8.2) | 0.897 |
| Precipitating event |  |  | 0.794 |
| HBV-reactivation | 183(88.4) | 85(87.6) |  |
| Spontaneous reactivation | 145(70) | 66(68) |  |
| NUC cessation | 26(12.6) | 15(15.5） |  |
| NUC resistance | 12(5.8) | 4（4.1） |  |
| Infection | 22(10.6) | 12（12.4） |  |
| Others | 2(1) | 0（0） |  |
| HBeAg positive | 86(41.5) | 38(39.2) | 0.696 |
| HBeAb positive | 133(64.3) | 65(67) | 0.639 |
| Lg(HBV-DNA) | 4(3,6) | 5(3,7) | 0.148 |
| Complications | | | |
| HE | 8(3.9) | 17(17.5) | <0.0001 |
| Ascites | 96(46.4) | 52(53.6) | 0.24 |
| GI Bleeding | 5(2.4) | 4(4.1) | 0.414 |
| bacterial infection | 24(11.6) | 18(18.6) | 0.102 |
| MELD | 22.0(19.6,24.4) | 24.5(21.2,28.1) | <0.0001 |
| MELDNa | 22.3(19.9,24.6) | 25.2(22.3,29.8) | <0.0001 |
| CLIF-C ACLF | 38.3(34.7,42.0) | 43.6(39.1,47.5) | <0.0001 |
| COSSH ACLF | 5.8(5.5,6.2) | 6.5(6.0,7.1) | <0.0001 |
| Laboratory data | | | |
| ALT (U/L) | 340(153,733) | 385(153,712) | 0.559 |
| AST (U/L) | 232(110,470) | 287(143,464) | 0.283 |
| TB (μmol/L) | 18.60(14.44,23.86) | 22.75(16.2,30.35) | <0.001 |
| ALP(U/L) | 135(115,163) | 140(122,168) | 0.06 |
| GGT(U/L) | 85(63,129) | 91(64,124) | 0.593 |
| Albumin (g/dL) | 32.0(29.3,34.8) | 31.5(29.8,34.8) | 0.799 |
| Sodium (mmol/L) | 138(136,140) | 138(135,139.5) | 0.034 |
| glucose（mmol/L） | 3.82(3.12,4.85) | 3.89(3.06,5.95) | 0.62 |
| Creatinine (μmol/L) | 0.75(0.63,0.87) | 0.72(0.62,0.85) | 0.588 |
| INR | 1.92(1.67,2.26) | 2.31(1.77,2.81) | <0.001 |
| Fibrinogen | 1.38(1.11,1.67) | 1.31(1.01,1.54) | 0.062 |
| D-Dimer | 1698(653,2640) | 2199(1490,3575) | <0.001 |
| WBC (10^9^/L) | 6(4.6,8.2) | 8(6,10) | <0.001 |
| Hemoglobin (g/L) | 132(120,146) | 138(120,149) | 0.151 |
| C-reactive protein | 11.5(7.85,15.75) | 12(8,19) | 0.874 |
| Platelet count (10^9^/L) | 109(83,145) | 116(75.5,136.5) | 0.553 |
| Ferritin (μg/L) | 2839(1662,4312) | 3552(1802,6356) | 0.015 |
| Alpha fetoprotein (μg/L) | 129.9(54.1,296.3) | 58.3(23.8,168.4) | <0.001 |

Data are expressed as mean ± SD or median with quartile range (P25, p75) or number of patients (percentages). ALP: phosphatase alkaline; ALT: alanine aminotransferase; AST: aspartate aminotransferase; BUN: blood urea nitrogen; CLIF-C ACLF: European Association for the Study of Chronic Liver Failure; COSSH-ACLF: Chinese Group on the Study of Severe Hepatitis B; GGT: gamma-glutamyl transpeptidase; GI: gastrointestinal; HE: hepatic encephalopathy; INR: international normalized ratio; MELD: Model for End-stage Liver Disease; NUC: nucleoside analogs; WBC: white blood count.
